# Supplementary material for: Constitutive signal bias mediated by the human GHRHR splice variant 1
Source: Proc Natl Acad Sci U S A. 2021 Oct 1;118(40):e2106606118. doi: 10.1073/pnas.2106606118 (PMC8501799; doi:10.1073/pnas.2106606118)
Supplement: Supplementary File [file pnas.2106606118.sapp.pdf]

PNAS
www.pnas.org

Supporting Information for

Constitutive signal bias mediated by the human GHRHR splice variant 1

Zhaotong Cong[1,2,11], Fulai Zhou[3,11], Chao Zhang[4,5,11], Xinyu Zou[6,11], Huibing Zhang[7], Yuzhe Wang[5,8], Qingtong Zhou[1], Xiaoqing Cai[3,8], Qiaofeng Liu[2], Jie Li[1], Lijun Shao[4,5], Chunyou Mao[7], Xi Wang[5,8], Jihong Wu[9], Tian Xia[6], Lihua Zhao[3], Hualiang Jiang[10], Yan Zhang[7], H. Eric Xu[3,*], Xi Cheng[10,*], Dehua Yang[3,5,8,*], Ming-Wei Wang[1,2,3,4,5,8,*]

[1]Department of Pharmacology, School of Basic Medical Sciences, Fudan University, Shanghai 200032, China; [2]School of Pharmacy, Fudan University, Shanghai 201203, China; [3]The CAS Key Laboratory of Receptor Research, Shanghai Institute of Materia Medica, Chinese Academy of Sciences, Shanghai 201203, China; [4]School of Life Science and Technology, ShanghaiTech University, Shanghai 201210, China; [5]University of Chinese Academy of Sciences, Beijing 100049, China; [6]School of Artificial Intelligence and Automation, Huazhong University of Science and Technology, Wuhan 430074, China; [7]Department of Biophysics and Department of Pathology of Sir Run Run Shaw Hospital, Zhejiang University School of Medicine, Hangzhou 310058, China; [8]The National Center for Drug Screening, Shanghai Institute of Materia Medica, Chinese Academy of Sciences, Shanghai 201203, China; [9]Eye and ENT Hospital, Fudan University, Shanghai 200031, China; [10]State Key Laboratory of Drug Research and Drug Discovery and Design Center, Shanghai Institute of Materia Medica, Chinese Academy of Sciences, 201203, Shanghai, China; [11]These authors contributed equally to this work.

*Correspondence: H. Eric Xu, Xi Cheng, Dehua Yang, Ming-Wei Wang

Email: eric.xu@simm.ac.cn, xicheng@simm.ac.cn, dhyang@simm.ac.cn, mwwang@simm.ac.cn

This PDF file includes:

Figures S1 to S9
Tables S1 to S4

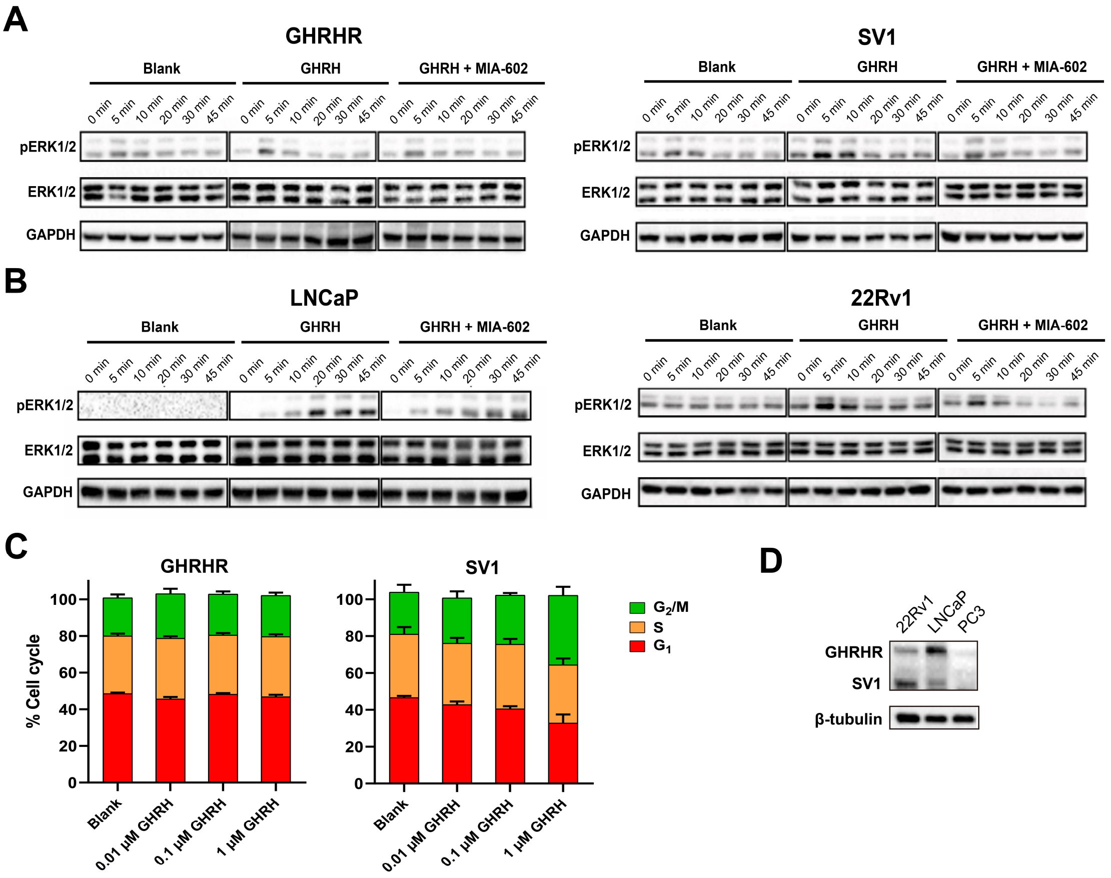

Figure S1. GHRH-induced pERK1/2 signaling mediated by SV1 and GHRHR and concurrent cell cycle change. (*A, B*) Representative time-course signaling of ERK1/2 monitored by immunoblotting of the total ERK1/2 and phosphorylated ERK1/2 (pERK1/2). The assay was initiated by 1 μM GHRH and inhibition was achieved by 4 μM MIA-602 in HEK293T cells expressing GHRHR or SV1 (*A*) and prostate cancer cell lines (*B*). (*C*) Cell distribution in G_1, S and G_2/M phases after treatment of different concentrations of GHRH. Data shown are means ± S.E.M. of at least three independent experiments (*n* = 3-5) performed in duplicate. (*D*) Expression of GHRHR and SV1 in prostate cancer cell lines.

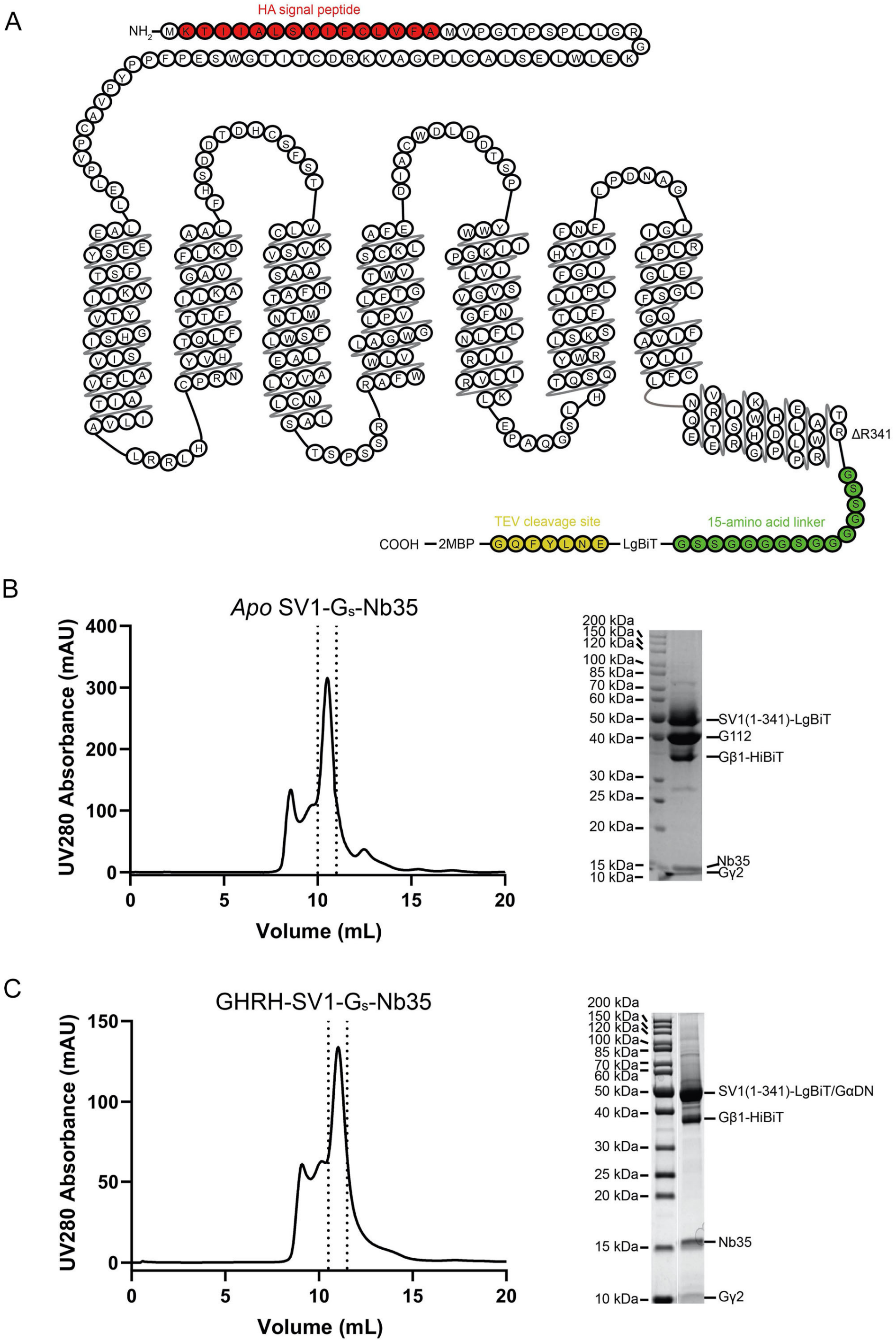

Figure S2. Purification and characterization of the SV1–G$_s$–Nb35 complex. (*A*) Schematic of the HA-SV1(1-341)-15AA-LgBiT-TEV-2MBP construct used in cryo-EM study. The HA signal peptide (red), 15-amino acid (AA) linker (green), Tev cleavage site (yellow) and R341 truncation site are highlighted and indicated. (*B*, *C*) Size-exclusion chromatography elution profile and corresponding SDS-PAGE gel of the *apo* SV1–G$_s$–Nb35 (*B*) and GHRH–SV1–G$_s$–Nb35 (*C*) complexes. G112 is an engineered Gα$_s$ protein.

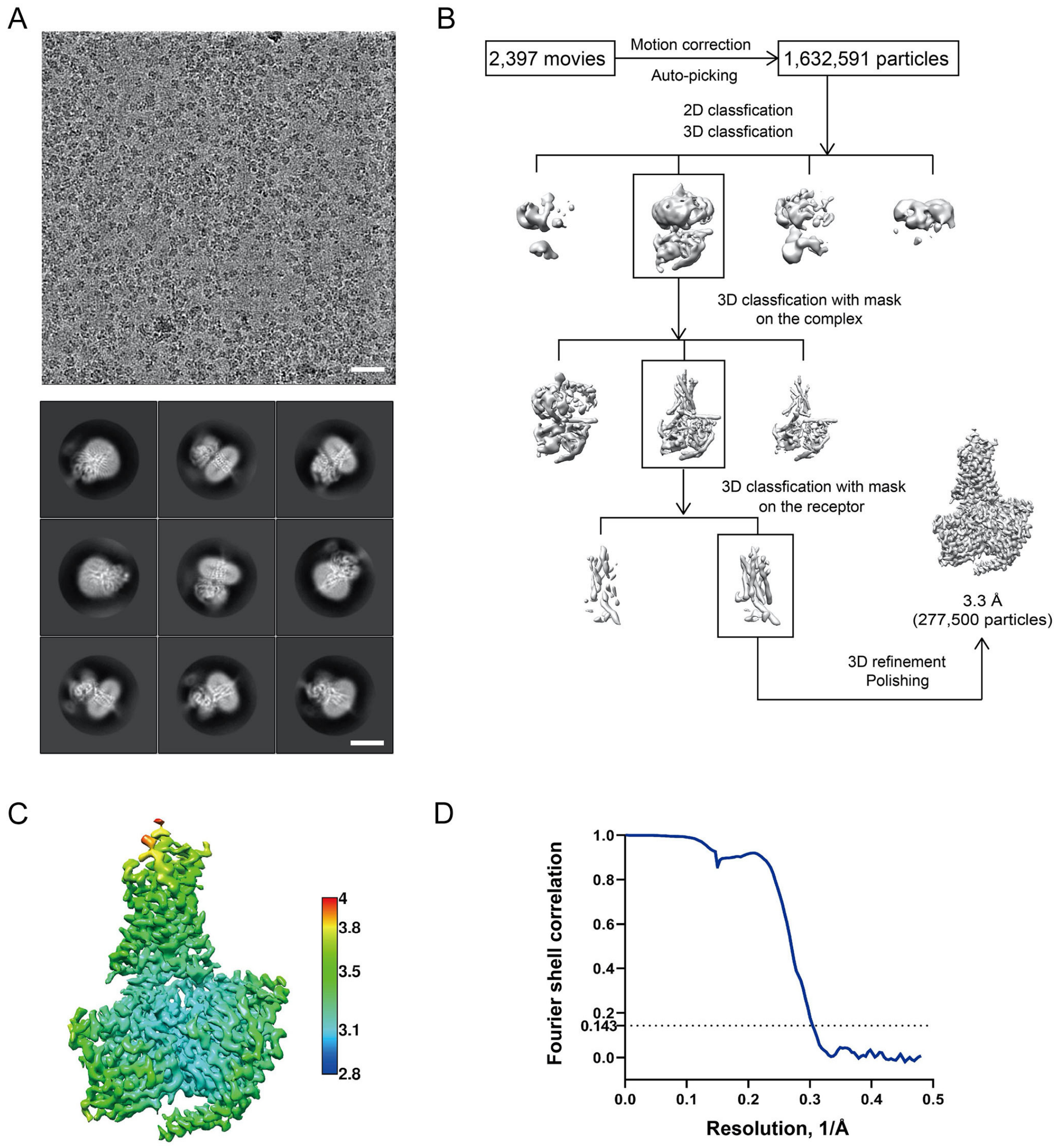

Figure S3. Cryo-EM data processing and validation of the GHRH–SV1–G_s–Nb35 complex. (*A*) Representative cryo-EM micrograph (scale bar: 40 nm) and two-dimensional class averages (scale bar: 5 nm). (*B*) Flow chart of cryo-EM data processing. (*C*) Local resolution distribution map of the complex. (*D*) Fourier shell correlation (FSC) curve of the overall refined receptor.

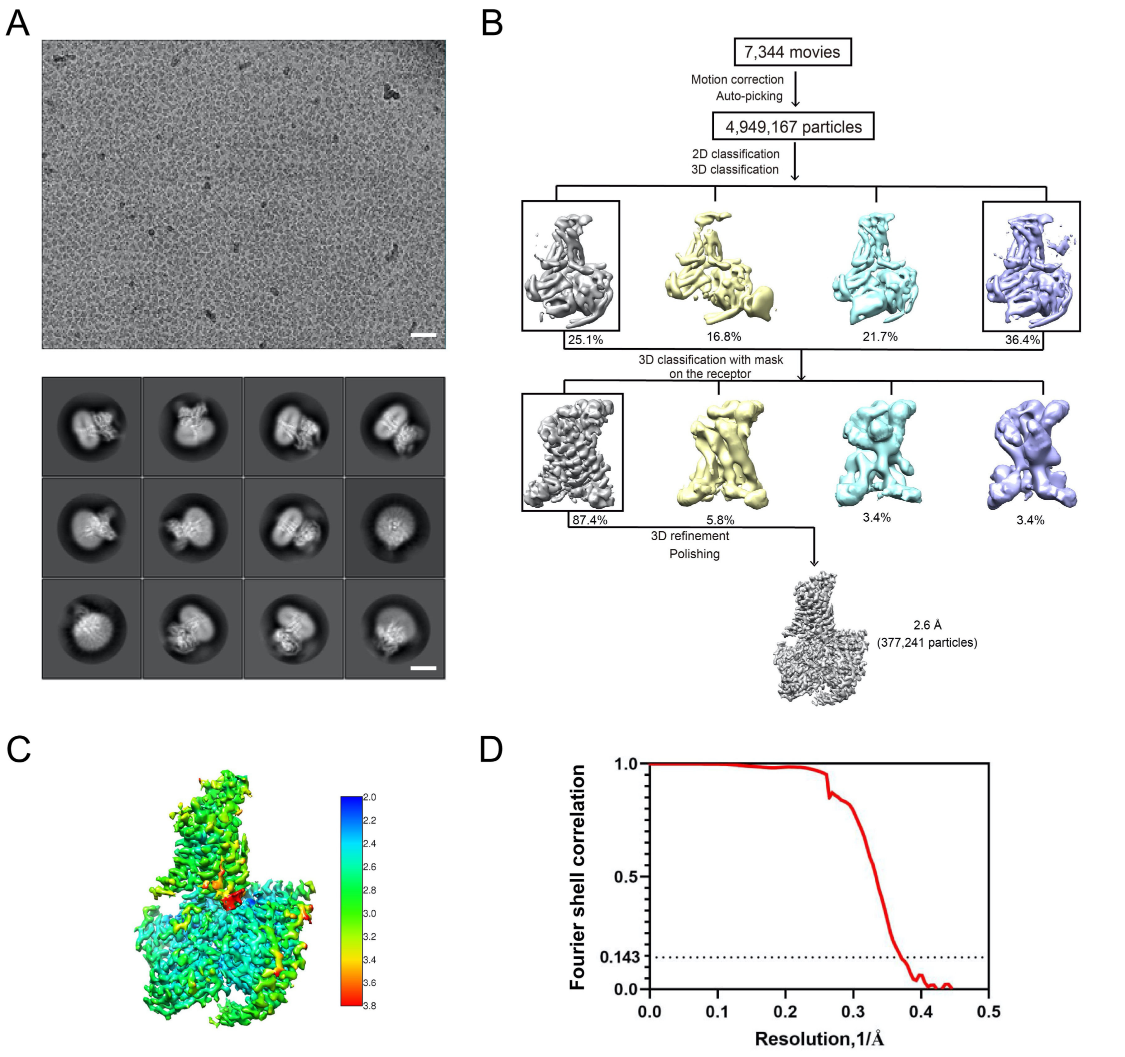

Figure S4. Cryo-EM data processing and validation of the *apo* SV1–G_s–Nb35 complex. (*A*) Representative cryo-EM micrograph (scale bar: 40 nm) and two-dimensional class averages (scale bar: 5 nm). (*B*) Flow chart of cryo-EM data processing. (*C*) Local resolution distribution map of the complex. (*D*) FSC curves of the overall refined receptor.

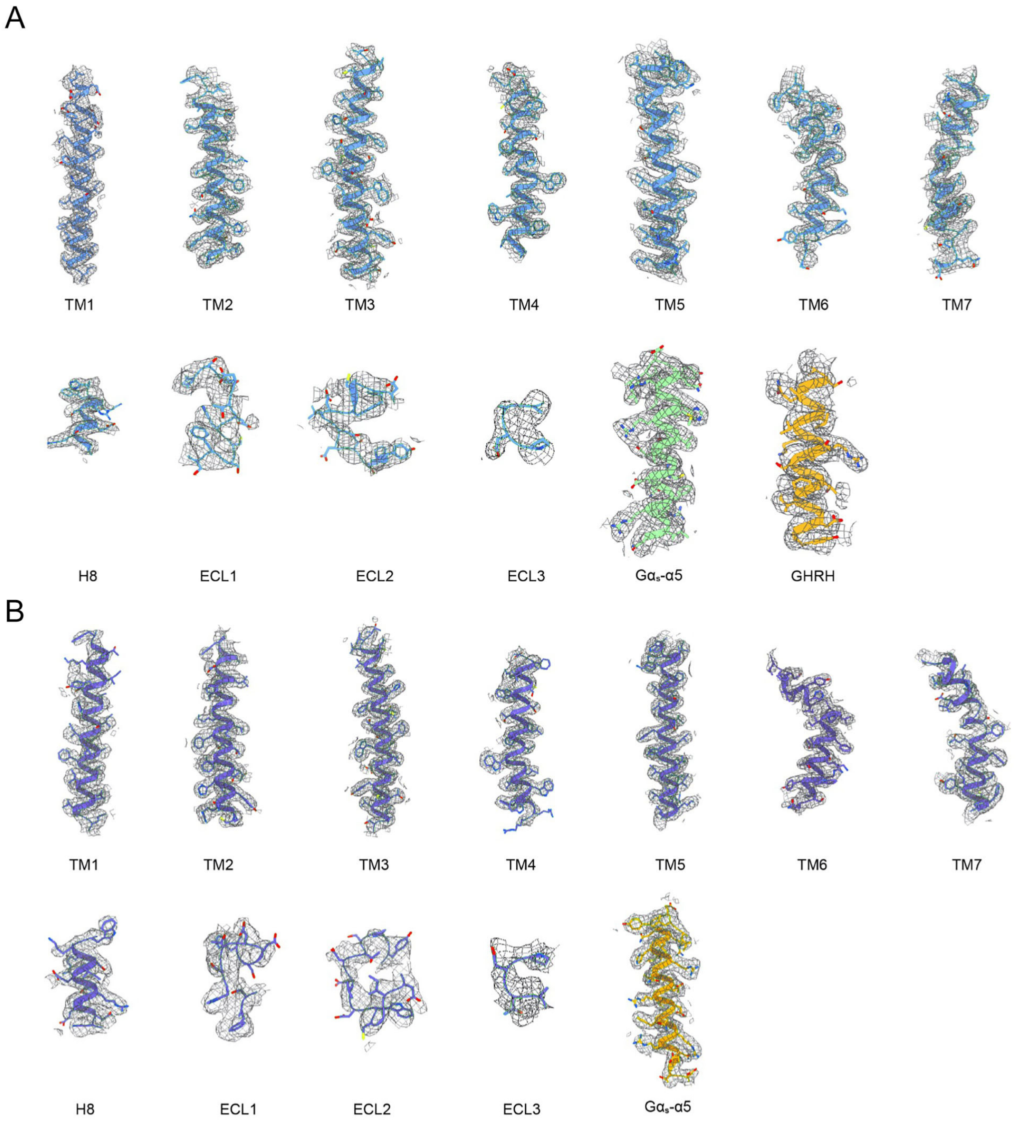

Figure S5. Cryo-EM density map of the GHRH–SV1–G_s and the *apo* SV1–G_s structures. (*A*) Cryo-EM density map and model of the GHRH–SV1–G_s structure are shown for all seven-transmembrane (TM) α-helices, ECLs 1-3, helix 8 (H8) of SV1, GHRH, Gα and helix α5. (*B*) Cryo-EM density map and model of the *apo* SV1–G_s structure are shown for all 7-TM α-helices, ECLs 1-3, helix 8 of SV1, Gα and helix α5.

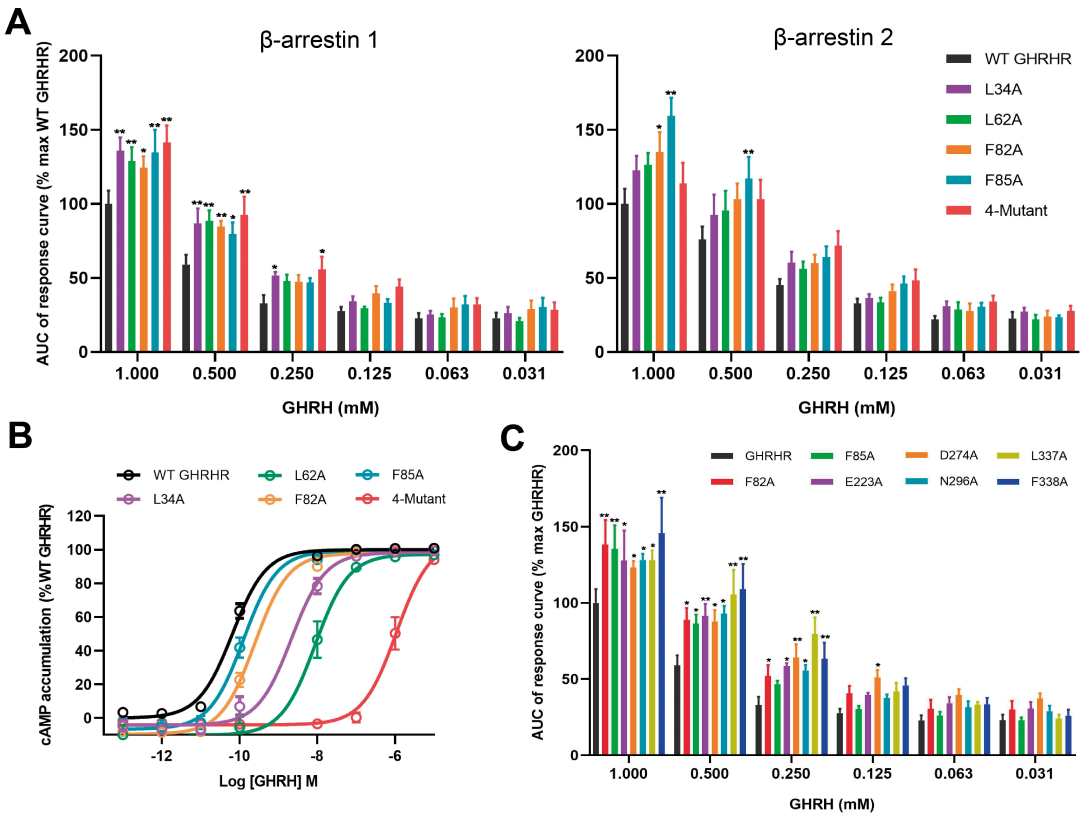

Figure S6. Residues of GHRHR responsible for biased signaling. (*A*) Mutations in the extracellular domain (ECD) reduced the cAMP response of GHRHR. (*B*) Mutations in the ECD affect β-arrestin 1/2 recruitment by GHRHR. 4-Mutant, single-point GHRHR mutation made simultaneously at 4 residues, L34A, L62A, F82A and F85A. (*C*) β-arrestin 1 recruitment by GHRHR and its mutants. Data shown are means ± S.E.M. of five independent experiments ($n = 5$) performed in quadruplicate or duplicate, respectively; $^*P < 0.05$, $^{**}P < 0.01$. WT, wild-type; max, maximum.

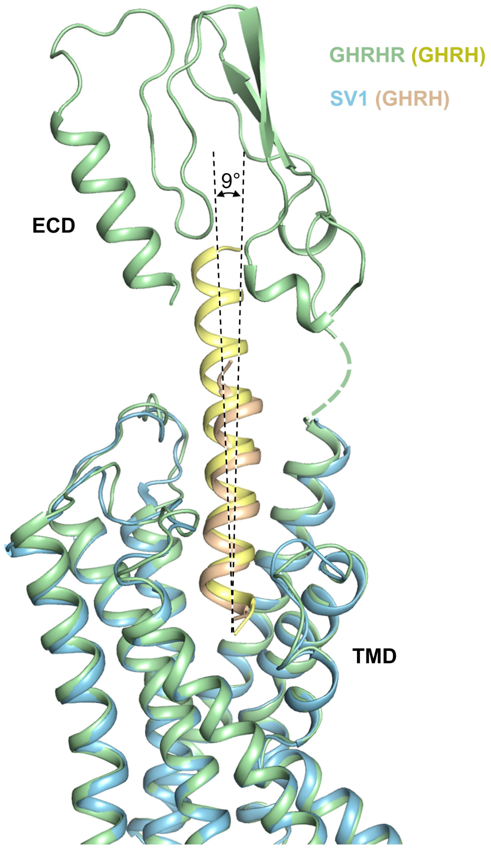

Figure S7. Comparison between the cryo-EM structures of GHRH–SV1–G_s and GHRH–GHRHR–G_s complexes at the extracellular side. Receptors and GHRH are shown in cartoon: GHRHR is colored in green, SV1 in blue, GHRH in wheat and yellow. G_s is omitted for clarity. ECD, extracellular domain; TMD, transmembrane domain.

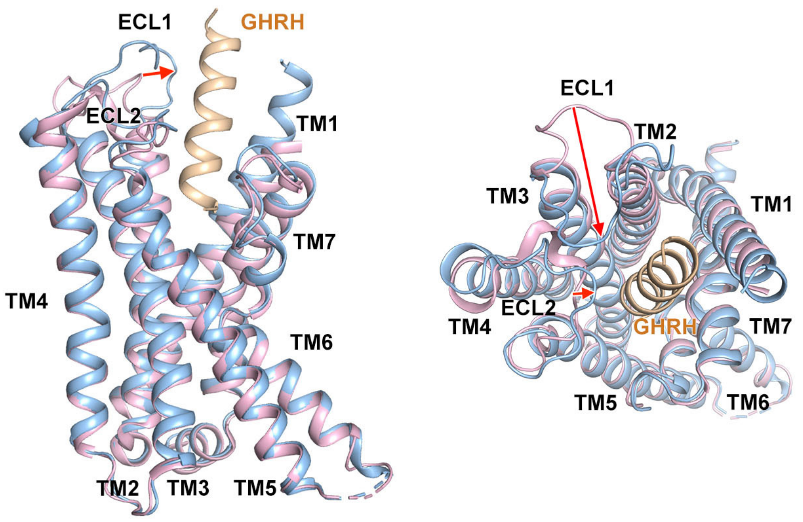

Figure S8. Comparison of the GHRH–SV1–G$_s$ complex structure with that of the *apo* SV1–G$_s$ complex. Both side (left) and top (right) views are displayed. Receptors and GHRH are shown in cartoon. In the GHRH–SV1–G$_s$ complex structure, SV1 is colored in blue and GHRH is in wheat. In the *apo* SV1–G$_s$ complex structure, SV1 is colored in pink. G$_s$ is omitted for clarity.

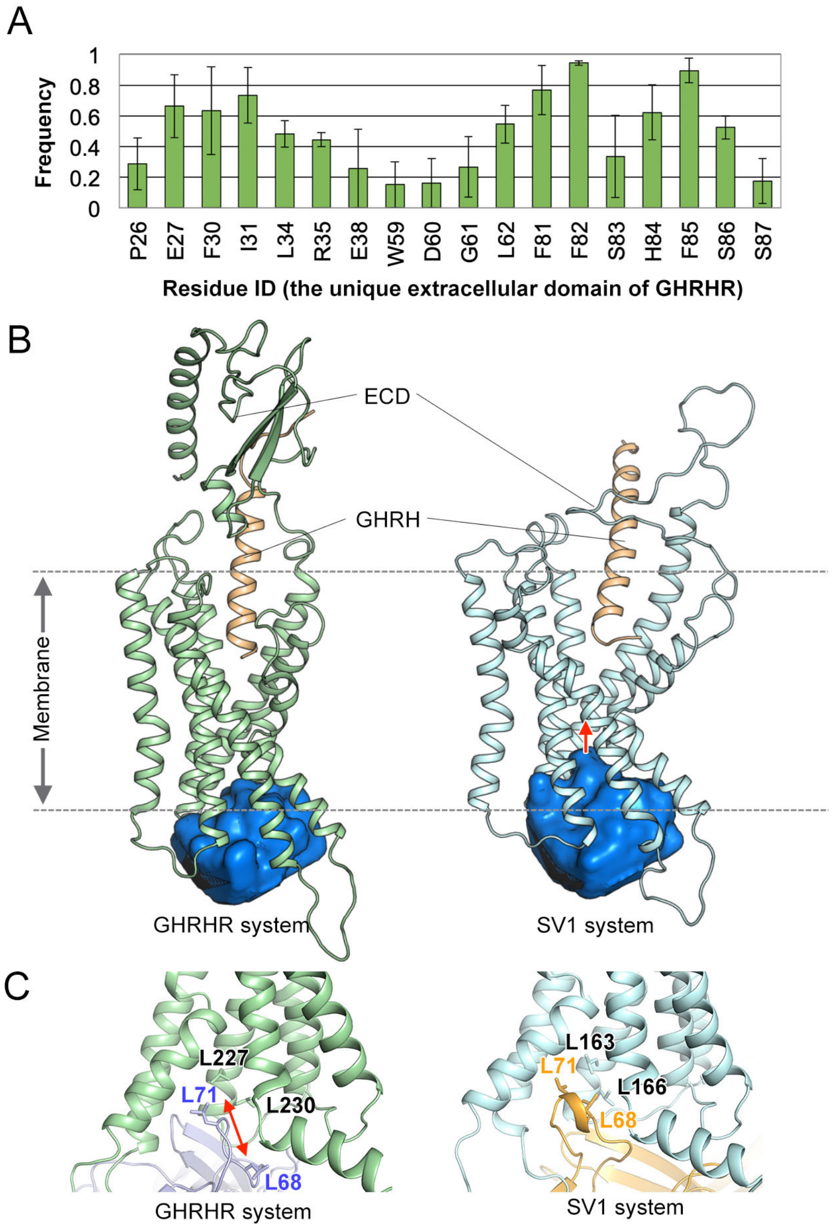

Figure S9. Extracellular and intracellular interactions of GHRHR and SV1. (*A*) Interacting frequency between an ECD residue of GHRHR and GHRH. The interacting frequency value indicates the stability of a particular residue-peptide interaction. A large interacting frequency implies a stable interaction. (*B*) Representative simulation snapshots from GHRHR system (left) and SV1 system (right). Receptors and GHRH are shown in cartoon: GHRHR is colored in green, SV1 in blue, GHRH in wheat. β-arrestin 1 is omitted for clarity. Arrestin-binding pockets are shown in surface depict. (*C*) A representative simulation snapshot showing key interactions of GHRHR (green) and SV1 (blue) at intracellular side. Key residues are shown as sticks.

Table S1. Effects of SV1 on GHRH-induced G_s activation.

Plasmid	pEC_{50}	E_{max} (% WT GHRHR)
GHRHR/G_s	8.17 ± 0.17	84.99 ± 3.32
SV1/G_s	5.63 ± 0.36*	56.67 ± 13.79*
Vector/G_s	NA	NA

G protein NanoBiT data were analyzed using a three-parameter logistic equation to determine pEC_{50} and E_{max} values. pEC_{50} is the negative logarithm of the molar concentration of agonist that induced half the maximal response. E_{max} is expressed as a percentage of GHRHR/G_s response. All values are means ± S.E.M. of five independent experiments (n = 5) conducted in duplicate. One-way ANOVA was used to determine statistical significance (*P < 0.05). WT, wild-type; NA, not active.

Table S2. Effects of residue mutation or truncation in the ECD of GHRHR on GHRH-induced cAMP accumulation.

Mutant	pEC_{50}	E_{max} (% WT GHRHR)	Cell surface expression (% WT GHRHR)	ΔLog (τ/K_A)
WT GHRHR	10.38 ± 0.07	100.50 ± 1.81	100	0 ± 0.05
SV1	6.43 ± 0.06***	104.85 ± 2.20	31.37 ± 6.83	-2.98 ± 0.04***
GHRHR Δ89	6.27 ± 0.06***	106.29 ± 2.48	49.46 ± 8.69	-3.28 ± 0.03***
GHRHR ΔECD	5.53 ± 0.07***	111.27 ± 3.65	209.26 ± 5.61	-4.55 ± 0.04***
GHRHR Δ32	6.55 ± 0.05***	105.06 ± 1.93	79.27 ± 3.78	-3.23 ± 0.04***
GHRHR Δ42	6.95 ± 0.04***	104.23 ± 1.53	91.80 ± 4.81	-2.86 ± 0.02***
GHRHR Δ52	6.62 ± 0.04***	105.63 ± 1.62	40.54 ± 5.0	-2.83 ± 0.03***
GHRHR Δ62	6.34 ± 0.05***	103.91 ± 2.19	31.30 ± 6.55	-3.05 ± 0.03***
GHRHR Δ72	6.44 ± 0.06***	104.35 ± 2.57	35.73 ± 7.58	-2.98 ± 0.04***
GHRHR Δ82	6.23 ± 0.06***	104.32 ± 2.51	31.60 ± 5.79	-3.14 ± 0.03***
GHRHR Δ92	6.25 ± 0.06***	105.98 ± 2.38	45.34 ± 5.32	-3.78 ± 0.03***
GHRHR Δ102	6.09 ± 0.05***	107.29 ± 2.15	121.17 ± 12.35	-3.55 ± 0.02***
GHRHR Δ112	5.93 ± 0.05***	106.27 ± 2.34	54.92 ± 7.98	-3.65 ± 0.02***
L34A	8.67 ± 0.11***	100.70 ± 2.23	94.27 ± 12.02	-0.78 ± 0.06***
L62A	8.06 ± 0.10***	97.99 ± 2.00	62.40 ± 5.34	-1.03 ± 0.04***
F82A	9.61 ± 0.49***	99.77 ± 1.36	102.74 ± 12.13	-0.53 ± 0.05***
F85A	9.91 ± 0.06**	100.24 ± 1.69	96.75 ± 10.38	-0.27 ± 0.04***
L34A-L62A-F82A-F85A	5.97 ± 0.09***	105.23 ± 5.52	77.50 ± 6.89	-4.07 ± 0.04***

cAMP accumulation data were analyzed using a three-parameter logistic equation to determine pEC_{50} and E_{max} values. pEC_{50} is the negative logarithm of the molar concentration of agonist that induced half the maximal response. E_{max} for mutants is expressed as a percentage of the WT GHRHR. Data were analyzed by nonlinear regression using the operational model equation to determine the logR values (logτ/K_A, *i.e.,* logarithm of the transduction ratio). τ is the efficacy value of the agonist and was corrected by cell surface expression of the receptor. K_A is dissociation constant. Changes in transduction ratio (ΔlogR) were calculated to determine the relative effectiveness of the mutants. All values are means ± S.E.M. of at least three independent experiments (n = 3-5) conducted in quadruplicate. Statistical analysis was carried out by comparing the control responses in the WT GHRHR. **, $P < 0.01$ and ***, $P < 0.001$, determined by one-way ANOVA.

Table S3. Cryo-EM data collection, model refinement and validation statistics.

Data collection and processing	GHRH-SV1-G_s-Nb35	SV1-G_s-Nb35
Magnification	130,000	130,000
Voltage (kV)	300	300
Electron exposure (e–/Å^2)	80	73
Defocus range (μm)	-1.2 to -2.2	-1.5 to -2.5
Pixel size (Å)	1.04	1.045
Symmetry imposed	C1	C1
Initial particle images (no.)	1,632,591	4,949,167
Final particle images (no.)	277,500	377,241
Map resolution (Å)	3.29	2.60
FSC threshold	0.143	0.143
Map resolution range (Å)	2.9-4.6	2.2-4.0
**Refinement**		
Initial model used (PDB code)	7CZ5	7CZ5
Model resolution (Å)	3.4	3.1
FSC threshold	0.5	0.5
Map sharpening B factor (Å^2)	-104.32	-82.56
Model composition		
Non-hydrogen atoms	8,139	8,047
Protein residues	1,029	1,019
B factors (Å^2)		
Protein	57.85	93.64
Root mean square deviation		
Bond lengths (Å)	0.004	0.004
Bond angles (°)	0.643	0.679
Validation		
MolProbity score	1.52	1.39
Clash score	4.39	3.50
Poor rotamers (%)	0.23	0.00
Ramachandran plot		
Favored (%)	95.63	96.31
Allowed (%)	4.37	3.69
Disallowed (%)	0.00	0.00

Table S4. Effects of residue mutation in the ligand-binding pocket of SV1 on GHRH-induced cAMP accumulation.

Mutant	pEC_{50}	E_{max} (% WT GHRHR)	Cell surface expression (% WT GHRHR)	ΔLog (τ/K_A)
GHRHR	10.83 ± 0.04	100	100	0.00 ± 0.09
SV1	6.38 ± 0.06	101.74 ± 2.28	47.56 ± 3.76	-3.93 ± 0.10
F62A	5.28 ± 0.08**	105.64 ± 4.54	58.47 ± 6.68	-4.41 ± 0.14***
V65A	5.33 ± 0.09*	106.08 ± 4.91	49.35 ±1.32	-4.08 ± 0.12
K66A	5.60 ± 0.08	106.82 ± 4.09	13.88 ± 2.07	-4.02 ± 0.11
Y69A	5.44 ± 0.09*	104.75 ± 4.29	36.69 ± 1.41	-3.81 ± 0.10
K118A	4.61 ± 0.12***	92.40 ± 7.25	57.63 ± 4.09	-4.51 ± 0.13***
S145A	6.34 ± 0.09	101.87 ± 3.50	37.13 ± 2.32	-4.22 ± 0.14**
H146A	5.07 ± 0.08***	101.06 ± 4.83	57.74 ± 7.57	-4.64 ± 0.11***
I225A	4.98 ± 0.12***	76.03 ± 5.48***	23.03 ± 2.05	-3.85 ± 0.13
L290A	5.58 ± 0.08	109.60 ± 3.90	34.40 ± 7.20	-4.56 ± 0.09***
L294A	5.32 ± 0.10*	106.26 ± 5.25	35.47 ± 1.85	-4.96 ± 0.12***

cAMP accumulation data were analyzed using a three-parameter logistic equation to determine pEC_{50} and E_{max} values. pEC_{50} is the negative logarithm of the molar concentration of agonist that induced half the maximal response. E_{max} for mutants is expressed as a percentage of the WT GHRHR. Data were analyzed by nonlinear regression using the operational model equation to determine the logR values ($\log\tau/K_A$, *i.e.*, logarithm of the transduction ratio). τ is the efficacy value of the agonist and was corrected by cell surface expression of the receptor. K_A is dissociation constant. Changes in transduction ratio (ΔlogR) were calculated to determine the relative effectiveness of the mutants. All values are means ± S.E.M. of four independent experiments (n = 4) conducted in quadruplicate. Statistical analysis was carried out by comparing the control responses in the WT-SV1. *, $P < 0.05$, **, $P < 0.01$ and ***, $P < 0.001$, determined by one-way ANOVA.
